# Identification of myeloid-derived suppressor cells in the synovial fluid of patients with rheumatoid arthritis: a pilot study

**DOI:** 10.1186/1471-2474-15-281

**Published:** 2014-08-19

**Authors:** Júlia Kurkó, András Vida, Tibor T Glant, Carla R Scanzello, Robert S Katz, Anjali Nair, Zoltán Szekanecz, Katalin Mikecz

**Affiliations:** Section of Molecular Medicine, Department of Orthopedic Surgery, Rush University Medical Center, 1735 W Harrison St, Cohn 712, Chicago, IL 60612 USA; Section of Rheumatology, Department of Internal Medicine, Rush University Medical Center, Chicago, IL 60612 USA; Division of Rheumatology, Perelman School of Medicine, University of Pennsylvania, Philadelphia, PA 19104 USA; Rheumatology Associates, Rush University Medical Center, Chicago, IL 60612 USA; Department of Rheumatology, Faculty of Medicine, University of Debrecen, 98 Nagyerdei St, H-4032 Debrecen, Hungary

**Keywords:** Rheumatoid arthritis, Myeloid-derived suppressor cells, T cells, Synovial fluid

## Abstract

**Background:**

Myeloid-derived suppressor cells (MDSCs) are a heterogeneous population of innate immune cells with a granulocyte-like or monocyte-like phenotype and a unique ability to suppress T-cell responses. MDSCs have been shown to accumulate in cancer patients, but recent studies suggest that these cells are also present in humans and animals suffering from autoimmune diseases. We previously identified MDSCs in the synovial fluid (SF) of mice with experimental autoimmune arthritis. The goal of the present study was to identify MDSCs in the SF of patients with rheumatoid arthritis (RA).

**Methods:**

RA SF cells were studied by flow cytometry using antibodies to MDSC cell surface markers as well as by analysis of cell morphology. The suppressor activity of RA SF cells toward autologous peripheral blood T cells was determined ex vivo. We employed both antigen-nonspecific (anti-CD3/CD28 antibodies) and antigen-specific (allogeneic cells) induction systems to test the effects of RA SF cells on the proliferation of autologous T cells.

**Results:**

SF from RA patients contained MDSC-like cells, the majority of which showed granulocyte (neutrophil)-like phenotype and morphology. RA SF cells significantly suppressed the proliferation of anti-CD3/CD28-stimulated autologous T cells upon co-culture. When compared side by side, RA SF cells had a more profound inhibitory effect on the alloantigen-induced than the anti-CD3/CD28-induced proliferation of autologous T cells.

**Conclusion:**

MDSCs are present among RA SF cells that are commonly regarded as inflammatory neutrophils. Our results suggest that the presence of neutrophil-like MDSCs in the SF is likely beneficial, as these cells have the ability to limit the expansion of joint-infiltrating T cells in RA.

**Electronic supplementary material:**

The online version of this article (doi:10.1186/1471-2474-15-281) contains supplementary material, which is available to authorized users.

## Background

Rheumatoid arthritis (RA) is an autoimmune disease characterized by inflammatory destruction of peripheral joints[1]. The involvement of autoreactive T cells in RA pathogenesis is supported by a genetic linkage between disease susceptibility and certain MHC class II (HLA-DR) molecules expressed by antigen-presenting cells[2], and by T-cell recognition of citrullinated autoantigens (autoAgs)[3]. Moreover, the presence of isotype-switched antibodies (Abs) against self IgG (i.e., rheumatoid factor) as well as against native and citrullinated self proteins in the majority of RA patients[4] is likely the result of help to Ab-producing B cells provided by autoreactive T helper (Th) cells[5]. T cells, belonging mainly to the Th1 and Th17 subsets, are also present in the rheumatoid joint and are believed to contribute greatly to local tissue damage[1, 6]. However, granulocytes (innate immune cells) constitute the major population of RA synovial fluid (SF) cells[1, 7]. Although SF granulocytes (mainly neutrophils) and monocytes can inflict considerable damage to joint structures through the release of proteolytic enzymes, pro-inflammatory cytokines, and other noxious substances[1], they may also do harm to joint-infiltrating T cells, thereby limiting the local expansion of these T cells.

Myeloid-derived suppressor cells (MDSCs) are cells of the innate immune system with a remarkable ability to suppress T-cell responses[8]. MDSCs are characterized by an “immature” phenotype on the basis of expression of CD33 (also present in myeloid precursors), and the absence or very low levels of HLA-DR[9, 10]. MDSCs also express the common myeloid marker CD11b, the α chain of the CD11b/CD18 leukocyte integrin heterodimer (also termed α_M_β_2_ integrin or Mac-1), which is found mainly on granulocytes, monocytes, and macrophages[11]. Indeed, MDSCs can be roughly categorized as granulocytic (CD15^+^ or CD66b^+^ cells showing polymorphonuclear morphology) and monocytic (CD14^+^ cells showing mononuclear morphology) subsets[8]. However, MDSCs belonging to these subsets (particularly granulocytic cells) exhibit a high degree of heterogeneity regarding nuclear morphology and the potency and the mechanism of immune suppression[12].

MDSCs were first identified in cancer patients and were shown to accumulate both in the vicinity of tumors and in peripheral blood[10, 13]. The survival and suppressive function of MDSCs are supported by tumor-produced myelopoietic growth factors including granulocyte macrophage colony-stimulating factor (GM-CSF), interleukin (IL)-6, granulocyte colony-stimulating factor (G-CSF) and others[14, 15], but some of these factors might also be produced at inflammatory sites[16–18].

Recent studies suggest that MDSCs are present at increased frequencies in the peripheral blood of patients with autoimmune diseases such as multiple sclerosis (MS)[19] and RA[20] as compared with healthy individuals. We previously identified MDSCs with a predominantly granulocytic phenotype in the SF of mice with proteoglycan-induced arthritis (PGIA, an autoimmune mouse model of RA)[21]. In this pilot study, we show that MDSCs are also present in the SF of RA patients.

## Methods

### Patients

Eleven RA patients undergoing therapeutic joint fluid aspiration at two clinics (Section of Rheumatology of the Department of Internal Medicine, and Rheumatology Associates) at Rush University Medical Center participated in the study. Informed consent was obtained from each of the participants. The 11 RA patients all donated SF, and 9 of them also donated blood. The specimens (SF and peripheral blood from RA patients and peripheral blood from a healthy volunteer) were collected through the Knee Injury and Arthritis Repository Study approved by the Institutional Review Board of Rush University Medical Center (Chicago, IL, USA). All patients had established RA according to the 2010 ACR/EULAR classification criteria[22] and substantial joint effusions requiring therapeutic aspiration. The mean age of the RA patients (9 females and 2 males) was 50.3 years (age range: 33–61 years).

### Analysis of cell surface marker expression and morphology of RA SF cells

Cells from the SF were pelleted by centrifugation (1000 rpm for 10 min at 4°C) and washed with sterile culture medium. Most of the SF samples had visible fibrin clots (formed in the syringe after joint aspiration). In these cases, clots and large cell aggregates were removed by passing the cell suspension through sterile 70 μm pore-size cell restrainer filters (BD Biosciences, San Diego, CA, USA). The cells were then counted and used for phenotypic, morphologic, and functional analyses without further separation. The reason for not separating MDSCs (which involves antibody-based positive selection for CD11b^+^ myeloid cells followed by sorting for CD33^+^HLA-DR^−^ MDSC-like cells) was that antibodies against CD11b (the α chain of the Mac-1 integrin, present in the majority of RA SF cells[1, 7]) had been reported to interfere with the function of myeloid cells including inhibition of T-cell responses[11, 23]. An aliquot of SF cells was immunostained and processed for flow cytometry. Before immunostaining, Fc receptors were blocked with purified human FcR inhibitor (eBioscience, San Diego, CA, USA) and then the cells were stained with fluorochrome-labeled monoclonal Abs (mAbs) against the following surface markers: CD11b, CD33, HLA-DR, CD14, and CD15 (from eBioscience or BioLegend, San Diego, CA, USA). Flow cytometry was performed using a BD FACS Canto II instrument, and data were analyzed with FACS Diva software (BD Flow Cytometry Systems, San Jose, CA, USA). For analysis of cell morphology, an aliquot of SF cells was spun onto glass slides, air dried, and stained with Wright-Giemsa solution (Sigma-Aldrich, St. Louis, MO, USA). Cytospin preparations were viewed and photographed using a Nikon Microphot light microscope (Nikon, Melville, NY, USA) equipped with a digital CCD camera (Coolsnap; RS Photometrics, Tucson, AR, USA).

### Cell isolation from human peripheral blood and suppression assays

Venous blood was collected in heparin-containing tubes, and peripheral blood mononuclear cells (PBMCs) were isolated on a Ficoll density gradient (GE Healthcare Life Sciences, Piscataway, NJ, USA) according to a standard protocol. After extensive washing, PBMCs were suspended in Dulbecco’s Modified Eagle Medium (DMEM) containing 10% fetal bovine serum (FBS; Hyclone, Logan, UT, USA). Viability (usually >95%) and cell counts for PBMCs as well as for SF cells were determined prior to culture. PBMCs were seeded in 96-well plates previously coated with anti-human-CD3 mAb (1 μg/well; BioLegend) at a density of 1 × 10^5^ cells per well in DMEM containing 10% FBS in the absence or presence of autologous (unseparated) SF cells at a ratio of 1:1. Background controls included PBMCs cultured in uncoated wells, and SF cells seeded in anti-CD3-coated wells. Anti-CD28 mAb (1 μg/well; BioLegend) was added in solution to cells in all anti-CD3-coated wells. The cells (4–6 wells/condition) were cultured for 4 days, and pulsed with [^3^H]thymidine (Perkin Elmer, Waltham, MA, USA) at 1 μCi/well for the last 18 hours of culture. Isotope incorporation (counts per minute: cpm) into dividing cells was measured in a MicroBeta scintillation counter (Perkin Elmer). As the actual cpm values varied from patient to patient, we calculated the ratios of T-cell proliferation in the presence of SF cells relative to positive control (i.e., in the absence of SF cells) after background correction.

We obtained sufficient numbers of both PBMCs and SF cells from 3 patients (RA #7, 8, and 9) to compare the effects of SF cells on anti-CD3/CD28-induced (non Ag-specific) and alloreactive (Ag-specific) proliferation of T cells side by side. To induce Ag-specific (allogeneic mixed leukocyte) response, RA PBMCs were co-cultured with allogeneic PBMCs from a HLA-mismatched healthy donor in the absence or presence of autologous SF cells at a RA PBMC:normal PBMC:RA SF cell ratio of 1:1:1. The allogeneic cells were treated for 30 min with Mitomycin C (40 μg/ml; Sigma-Aldrich) prior to co-culture to inhibit cell division, and proliferation of autologous (RA) T cells was determined on day 5 on the basis of [^3^H]thymidine incorporation. In this case, cmp values of Mitomycin C-treated allogeneic PBMCs (cultured alone) were included in the background controls.

Isotope incorporation (cpm values) by the cells of this group of 3 RA patients was reasonably similar when the cells were stimulated with anti-CD3/28 or with allogeneic PBMCs or cultured alone. This allowed us to compare the background-corrected cpm values as well as the proliferation ratios under the two different conditions of in vitro stimulation.

### Statistical analysis

Descriptive statistics was employed to determine the means ± SEM and 95% confidence intervals (95% CI). The parametric paired t test and the nonparametric Wilcoxon matched-pairs signed rank test were used (as appropriate) to detect statistically significant (p < 0.05) differences in the cpm values and in the proliferation ratios of RA PBMCs under the different culture conditions. Statistical analysis of data was performed using GraphPad Prism 6 program (GraphPad Software, La Jolla, CA, USA).

## Results

### Cells with phenotype and morphology resembling MDSCs are present in the SF of RA patients

**Figure 1:**
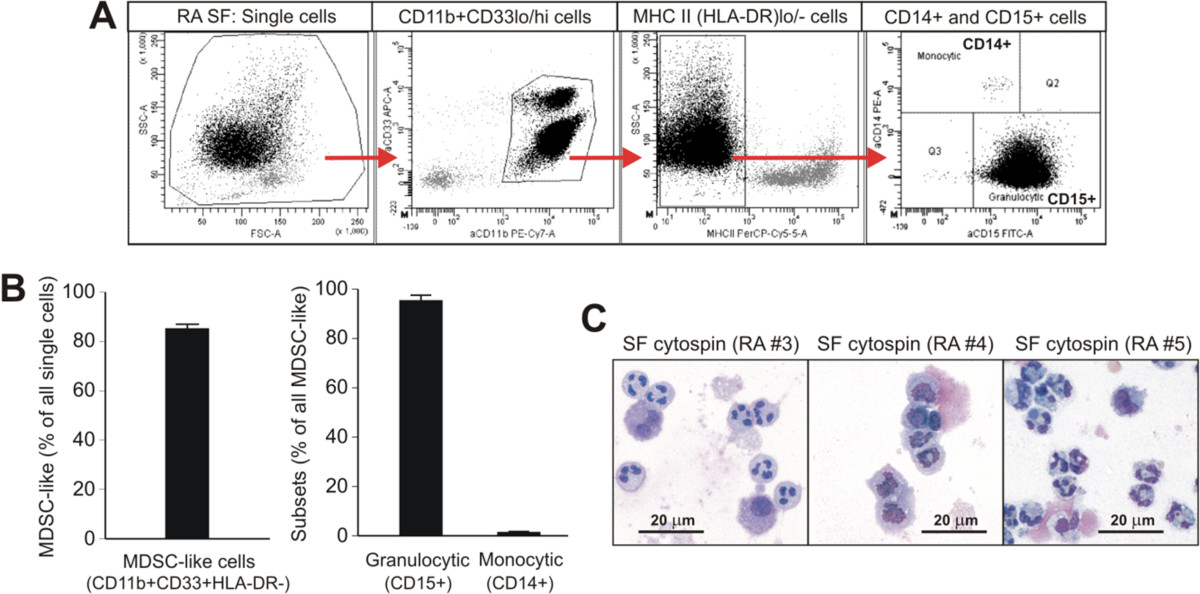
**Presence of cells with myeloid-derived suppressor cell (MDSC)-like phenotype and morphology in synovial fluid (SF) from rheumatoid arthritis (RA) patients. (A)** Flow cytometry profile of RA SF cells using a combination of antibodies against the common myeloid marker CD11b, the “immature” myeloid cell marker CD33, MHC class II (HLA-DR), the monocytic subset marker CD14, and the granulocytic subset marker CD15 (gating strategy is indicated by red arrows). The example shown (1 of 11 RA SF samples with similar profiles) demonstrates the dominance of CD11b^+^CD33^lo/hi^HLA-DR^lo/-^CD14^−^CD15^+^ (granulocytic) MDSC-like cells in RA SF. **(B)** Using the same gating strategy on the 11 RA SF samples, the mean frequency of MDSC-like myeloid cells was 85.03% (range: 76.1-97.9%) among the SF cells (left-side graph). The granulocytic subset represented 95.2% (range: 72.9-99.7%) and the monocytic subset represented 1.3% (range: 0.1-5.3%) of the MDSC-like SF cell population (bars in right-side graph). The data shown are the means ± SEM. **(C)** The morphology of SF cells in Wright-Giemsa-stained cytospin preparations from 3 RA patients also indicated the dominance of the polymorphonuclear granulocytic subset, although the shape of the nuclei of these neutrophil-like cells varied among the patients.

Screening for MDSC-like cells was carried out by flow cytometry using SF samples collected from 11 RA patients. We employed a combination of mAbs to MDSC cell surface markers including the common myeloid marker CD11b, the “immature” myeloid cell marker CD33, MHC II (HLA-DR), the monocytic MDSC subset marker CD14, and the granulocytic subset marker CD15[8–10]. As demonstrated by a representative sample, CD11b^+^CD33^+^HLA-DR^lo/-^CD14^−^CD15^+^ granulocytic MDSC-like cells were the predominant cell type in RA SF, but a small population of the CD11b^+^CD33^+^HLA-DR^lo/-^CD14^+^CD15^−^ monocytic subset was also present (Figure 1A). MDSC-like cells constituted ~85% of all SF cells (Figure 1B, left-side graph) and ~95% of these MDSC-like cells belonged to the granulocytic subset (Figure 1B, first bar in the right-side graph) in the samples of the 11 RA patients tested. Despite very similar cell surface marker expression profile in all RA SF samples (Figure 1A-B), the nuclear morphology of the cells varied among the patients, although the dominance of the polymorphonuclear (neutrophil-like) granulocytic subset was evident (Figure 1C).

### RA SF cells significantly suppress the anti-CD3/CD28-induced proliferation of autologous T cells

**Figure 2:**
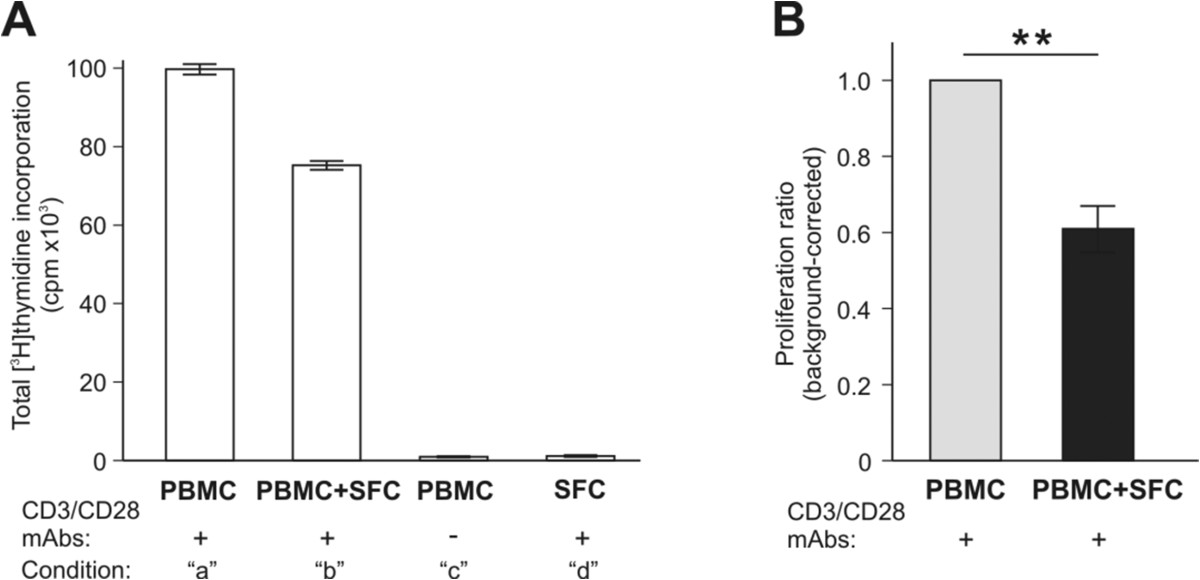
**Suppression of anti-CD3/CD28-induced polyclonal proliferation of autologous peripheral blood T cells by RA SF cells. (A)** Peripheral blood mononuclear cells (PBMC) stimulated with anti-CD3/CD28 monoclonal antibodies (Abs) were cultured in the absence (condition “a”) or presence (“b”) of SF cells (SFC) from the same RA patient (RA #3). Background controls included PBMCs cultured without anti-CD3/CD28 Abs (“c”) and SF cells cultured with anti-CD3/CD28 Abs (“d”). The results shown are the means ± SEM of isotope incorporation (counts per minute, cpm) by the proliferating cells (6 wells per condition). As the actual cpm values at all of the listed conditions varied from patient to patient, we calculated the proliferation ratio (with background correction) for each RA patient using the formula: [b − (c + d)]/(a − c), where the positive control (a - c) was set to 1. **(B)** As indicated by the proliferation ratios, the anti-CD3/CD28-induced proliferation of autologous PBMCs (light gray bar) was significantly suppressed in the presence of SF cells (black bar). The results shown are the means ± SEM of background-corrected proliferation ratios (**p < 0.01; Wilcoxon matched-pair signed rank test; n = 9 patients).

Lechner et al.[24] reported that monocytic MDSCs, generated in vitro from human PBMCs in the presence of GM-CSF and IL-6, were able to suppress the anti-CD3/CD28-induced proliferation of autologous T cells. To determine whether the MDSC-like cells that we identified in RA SF, indeed, had suppressive properties, we stimulated T cells (present in PBMC) with anti-CD3/CD28 mAbs in the absence and presence of SF cells from the same patients. Anti-CD3/CD28-stimulated T cells in PBMC proliferated less well in the presence than in the absence of autologous SF cells as indicated by the difference in total [^3^H]thymidine incorporation (Figure 2A, condition “a” versus condition “b”). Isotope incorporation (cpm) by unstimulated PBMCs or anti-CD3/CD28-treated SF cells (Figure 2A, conditions “c” and “d”, respectively, as background controls) was low, but still detectable. We tested PBMCs and SF cells from a total of 9 RA patients in the same in vitro system, and found that SF cells from all of these patients suppressed anti-CD3/CD28-induced cell proliferation. Since total isotope incorporation by PBMCs and PBMC-SF cell co-cultures as well as background cpm values (conditions “c” and “d”) varied from patient to patient, we calculated the background-corrected proliferation ratios for the 9 patients tested. As shown in Figure 2B, the SF cell-mediated suppression of anti-CD3/CD28-induced T-cell proliferation was statistically significant (p = 0.0039, 95% CI = 0.4682-0.7495).

### SF cells from the same RA patients are more potent in suppressing the Ag-specific than the anti-CD3/CD28-induced proliferation of autologous T cells

**Figure 3:**
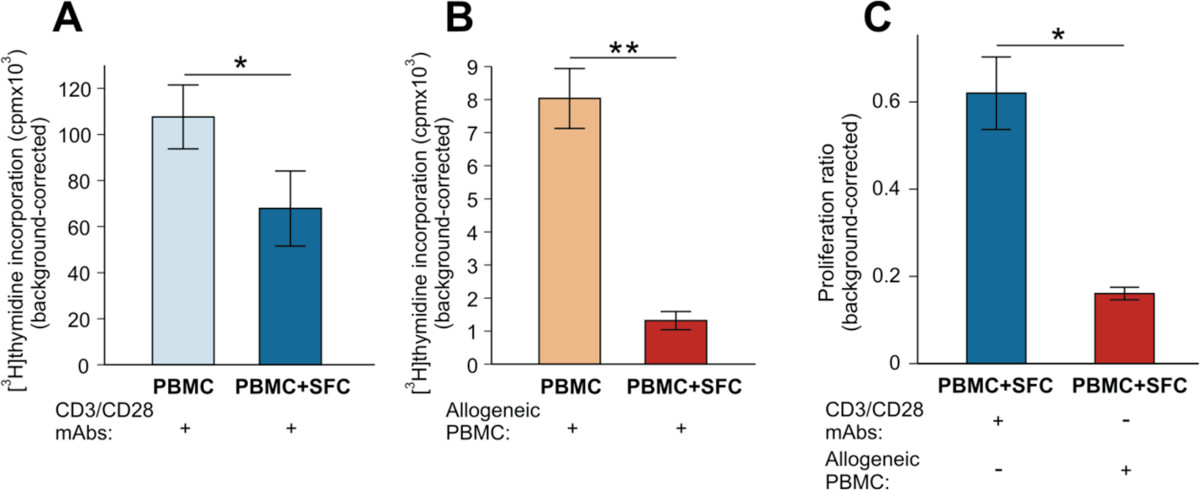
**Comparison of the suppressive effects of RA SF cells on the anti-CD3/CD28-induced and alloantigen-induced proliferation of autologous T cells. (A)** Isotope incorporation (cpm) by anti-CD3/CD28 Ab-stimulated PBMCs (light blue bar) was moderately, but significantly reduced in the presence (dark blue bar) of SF cells (SFC) from 3 RA patients (RA #7, 8, 9). **(B)** Allogeneic PBMC-induced proliferation of PBMCs from the same 3 patients (orange bar) was greatly reduced in the presence of autologous SF cells (dark red bar). The data shown in panels **A** and **B** are the means ± SEM of background-corrected cpm values (4 wells per condition) (*p < 0.05, **p < 0.01; Paired t test). **(C)** Comparison of the ratios of anti-CD3/CD28-induced (dark blue bar) and allogeneic PBMC-induced (dark red bar) proliferation of RA PBMCs in the presence of autologous SFC in the 3 RA patients (cpm values shown in panels **A** and **B**) indicated that SF cells from the same patients were more efficient in suppressing the allogeneic PBMC-induced than the anti-CD3/CD28-induced proliferation of autologous T cells. The data are the means ± SEM of background-corrected proliferation ratios (*p < 0.05; Paired t test).

We reported previously that MDSCs present in the SF of the arthritic joints of mice with PGIA potently suppressed Ag (human PG)-induced T-cell proliferation, but were ineffective against anti-CD3/CD28-induced proliferation[21]. In this study, using allogeneic PBMC as a source of Ag, we directly compared the effects of RA SF cells on the anti-CD3/CD28-induced versus alloAg-induced proliferation of autologous T cells obtained from the same patients. Side-by-side comparison of cell cultures of these RA patients (RA #7, 8, 9) demonstrated significant (p = 0.0389) but moderate activity of SF cells in inhibiting the robust proliferation of anti-CD3/CD28-stimulated autologous T cells (Figure 3A), and also significant (p = 0.0087) and more effective suppression of the Ag-induced (and more modest) proliferation of the same T cells (Figure 3B). Since the SF cell populations from the same RA patients exhibited significantly (p = 0.0413) different degrees of suppression under the two different culture conditions (Figure 3C), these results also suggested that inhibition of T-cell proliferation was not simply due to cytotoxic substances released from degranulating, dying, or apoptotic SF cells upon culture.

## Discussion

MDCSs have been gaining increasing attention in recent years as important modulators of adaptive immune responses in various diseases[8]. In cancer patients, accumulation of MDSCs around the tumors as well as at the periphery can be detrimental, as these cells suppress tumor Ag-specific T cells, thus weakening anti-tumor immunity[13, 25]. On the contrary, MDSC-mediated suppression of T-cell responses can be beneficial in pathologic conditions characterized by the unopposed activation of the adaptive immune system such as organ transplant rejection or autoimmune diseases[12]. Indeed, adoptive transfer of MDSCs in mouse models of human autoimmune disorders, including MS[19], type I diabetes[26], and RA[27], was followed by reduction in disease severity in the MDSC recipient mice.

Jiao et al.[20] reported increased frequency of MDSC-like cells in the blood of patients with RA as compared with healthy individuals, and also found a negative correlation between the frequencies of circulating MDSC-like and Th17 cells in RA patients. Unfortunately, MDSC-like cells were defined by phenotypic marker expression only, and the suppressor activity of these cells toward T cells was not tested in that study[20]. For the first time to our knowledge, here we show that MDSC-like cells are also present in the SF of RA patients. These cells are true MDSCs, as they are capable of suppressing the ex vivo induced proliferation of autologous T cells.

With regard to phenotype, we have found that the majority of MDSC-like RA SF cells belongs the granulocytic CD11b^+^CD33^+^HLA-DR^lo/-^CD14^−^CD15^+^ subset with neutrophil morphology; only a very small population of the CD11b^+^CD33^+^HLA-DR^lo/-^CD14^+^CD15^−^ monocytic subset could be identified in the patients’ SF samples. There is an ongoing debate about an association between phenotype and function, as both granulocytic and monocytic MDSCs have been reported to exhibit immune suppression in a disease- and tissue site-dependent manner[12, 28]. Moreover, within the granulocytic subset, suppressive cells have been identified among both “immature” neutrophils (with band-shaped nuclei) and mature neutrophils (with segmented or even hyper-segmented nuclei) in humans[29]. As we described earlier, SF cells harvested from the arthritic joints of mice with PGIA were also dominated by granulocytic cells with neutrophil morphology, and these SF cells retained their immune suppressive potential after removal of the minor monocytic MDSC subset[21]. In the collagen-induced mouse model of RA, granulocytic MDSCs isolated from the spleens of arthritic mice suppressed T-cell proliferation in vitro, and reduced the severity of joint inflammation upon adoptive transfer in vivo[27]. Taken together, these observations and our findings described in this study suggest that immune suppressive cells with the phenotypic and morphologic characteristics of neutrophils are present in the SF of RA patients.

Similar to SF cells collected from mice with PGIA[21], we found that SF cells from RA patients were much more potent in suppressing Ag-specific than anti-CD3/CD28-induced proliferation of autologous T cells. However, unlike mouse SF cells, SF MDSCs from RA patients were also able to exert a significant inhibitory effect on the vigorous proliferation of anti-CD3/CD28-stimulated T cells. These observations implicate SF MDSCs as non-selective suppressors of T-cell expansion, and also suggest that the difference in suppressive potency observed in the Ag-specific versus non-specific systems might simply be due to the difference in the magnitude of the response of T cells to these stimuli.

The mechanisms of MDSC-mediated suppression include depletion of L-arginine by arginase-1, synthesis of nitric oxide (NO) by inducible NO synthase, and production of various oxygen radicals[8], all of which can have negative effects on the cell cycle and CD3-related signaling in T cells[29]. We found that the primary mechanism of immune suppression by mouse granulocytic SF cells involved NO production[21]. The limited amount of patient samples available for this study did not allow us to investigate the suppressive mechanisms employed by RA SF cells. However, elevated concentrations of nitrite (formed from NO) have been reported in the SF of RA patients[30], suggesting the possibility that NO production is one of the mechanisms SF MDSCs use to suppress T-cell proliferation.

Myelopoiesis-supporting factors such as GM-CSF, G-CSF, and IL-6 have been implicated in the induction and survival of MDSCs[8, 17, 24, 31]. Notably, these growth factors are present at high levels in the SF of RA patients[18], thereby providing a milieu in which MDSCs can thrive. On the other hand, the widely observed “hypo-responsiveness” of RA SF T cells to mitogenic stimuli (as compared to the normal responsiveness of blood T cells from the same patient)[1, 32] might, at least in part, be related to the long-term exposure of T cells to MDSCs within the joint exudate. Moreover, although CD4^+^CD25^+^FoxP3^+^ regulatory T cells might be present in RA SF[5, 33], the inflammatory environment greatly reduces the capacity of these regulatory T cells to inhibit the activity and expansion of effector T cells ex vivo or within the joint[5, 34].

In the collagen-induced mouse model of RA, intravenous (systemic) transfer of spleen-derived MDSCs was followed by a decrease in the number of CD4^+^ T cells and reduced arthritis severity in the recipient mice[27]. Conversely, in vivo depletion of MDSCs prevented the spontaneous resolution of joint inflammation[27]. We propose that MDSCs present in RA SF, while inflicting collateral damage to joint tissues, also serve as negative regulators of local T-cell expansion in an attempt to break the vicious cycle of autoimmunity and inflammation.

## Conclusions

In this study, we show for the first time that MDSCs are present in the SF of RA patients. The majority of RA SF MDSCs exhibits neutrophil phenotype and morphology, similarly to MDSCs we identified earlier in the SF of mice with autoimmune arthritis. The suppression mediated by RA SF cells appears to be non-selective as these MDSCs potently suppress both the anti-CD3/CD28 Ab-induced and alloAg-induced proliferation of autologous blood T cells. We suggest that RA SF MDSCs are able to limit the expansion of joint-infiltrating (and most likely pathogenic) T cells. This pilot study represents the first step of investigations into the role of MDSCs (present in the inflamed joints and perhaps at other sites) in controlling autoimmune T-cell responses in RA.

## Authors’ original submitted files for images

Below are the links to the authors’ original submitted files for images. Authors’ original file for figure 1 Authors’ original file for figure 2 Authors’ original file for figure 3

## Abbreviations

Ab: Antibody
Ag: Antigen
CI: Confidence interval
cpm: Counts per minute
DMEM: Dulbecco’s modified Eagle medium
FBS: Fetal bovine serum
G-CSF: Granulocyte-colony stimulating factor
GM-CSF: Granulocyte/macrophage-colony stimulating factor
HLA-DR: Human leukocyte antigen-DR
IL-6: Interleukin-6
mAb: Monoclonal antibody
MDSC: Myeloid-derived suppressor cell
MHC II: Major histocompatibility complex class II
MS: Multiple sclerosis
NO: Nitric oxide
PBMC: Peripheral blood mononuclear cell
PG: Proteoglycan (cartilage aggrecan)
PGIA: PG-induced arthritis
RA: Rheumatoid arthritis
SEM: Standard error of the mean
SF: Synovial fluid
SFC: SF cell
Th: T helper.
