# Supplementary figures and images for: Identification of myeloid-derived suppressor cells in the synovial fluid of patients with rheumatoid arthritis: a pilot study
Source: BMC Musculoskelet Disord. 2014 Aug 19;15:281. doi: 10.1186/1471-2474-15-281 (PMC4152562; doi:10.1186/1471-2474-15-281)

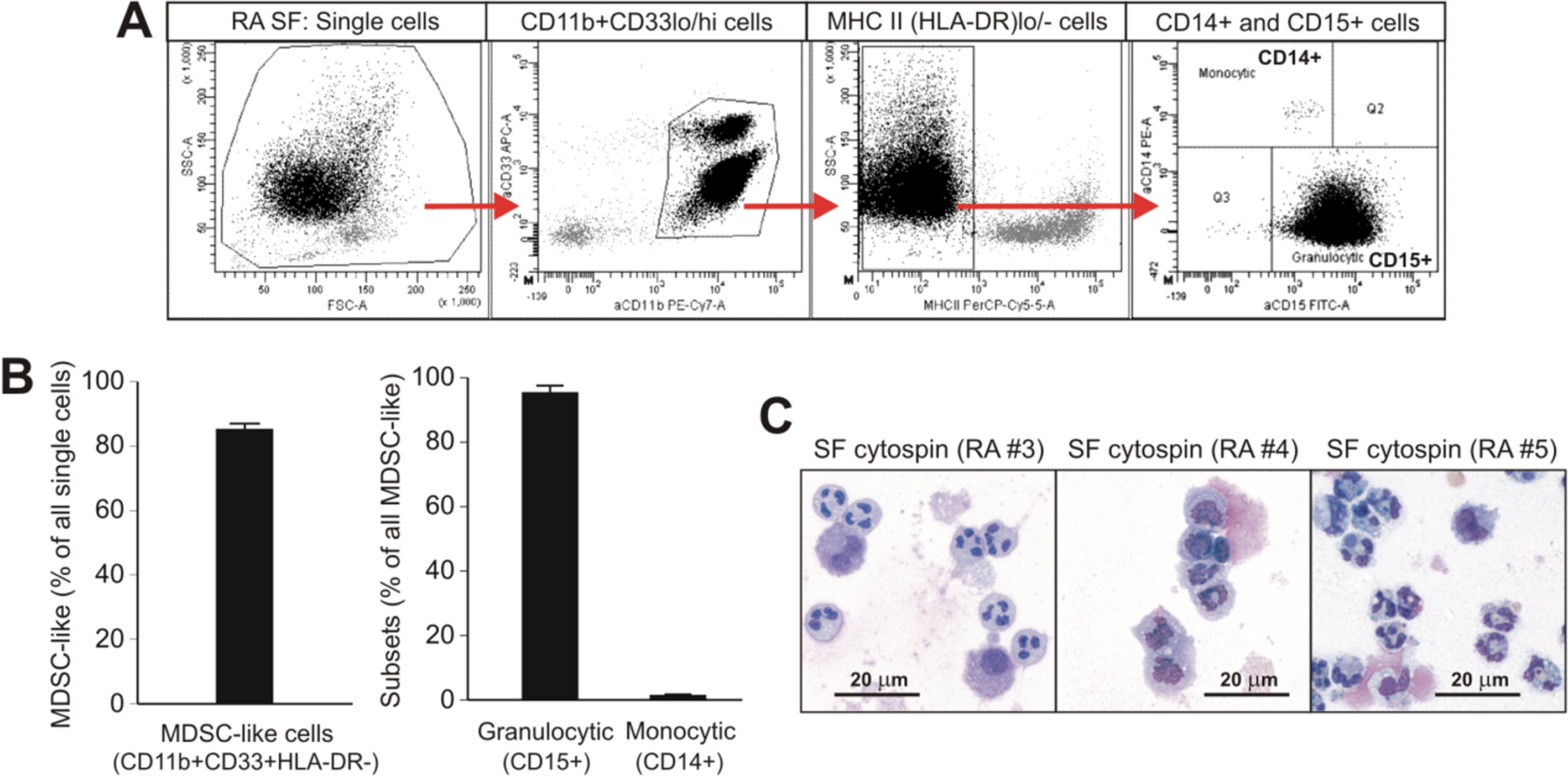

Supplement: Supplementary file 1 — Authors’ original file for figure 1 [file 12891_2014_2228_MOESM1_ESM.tif]

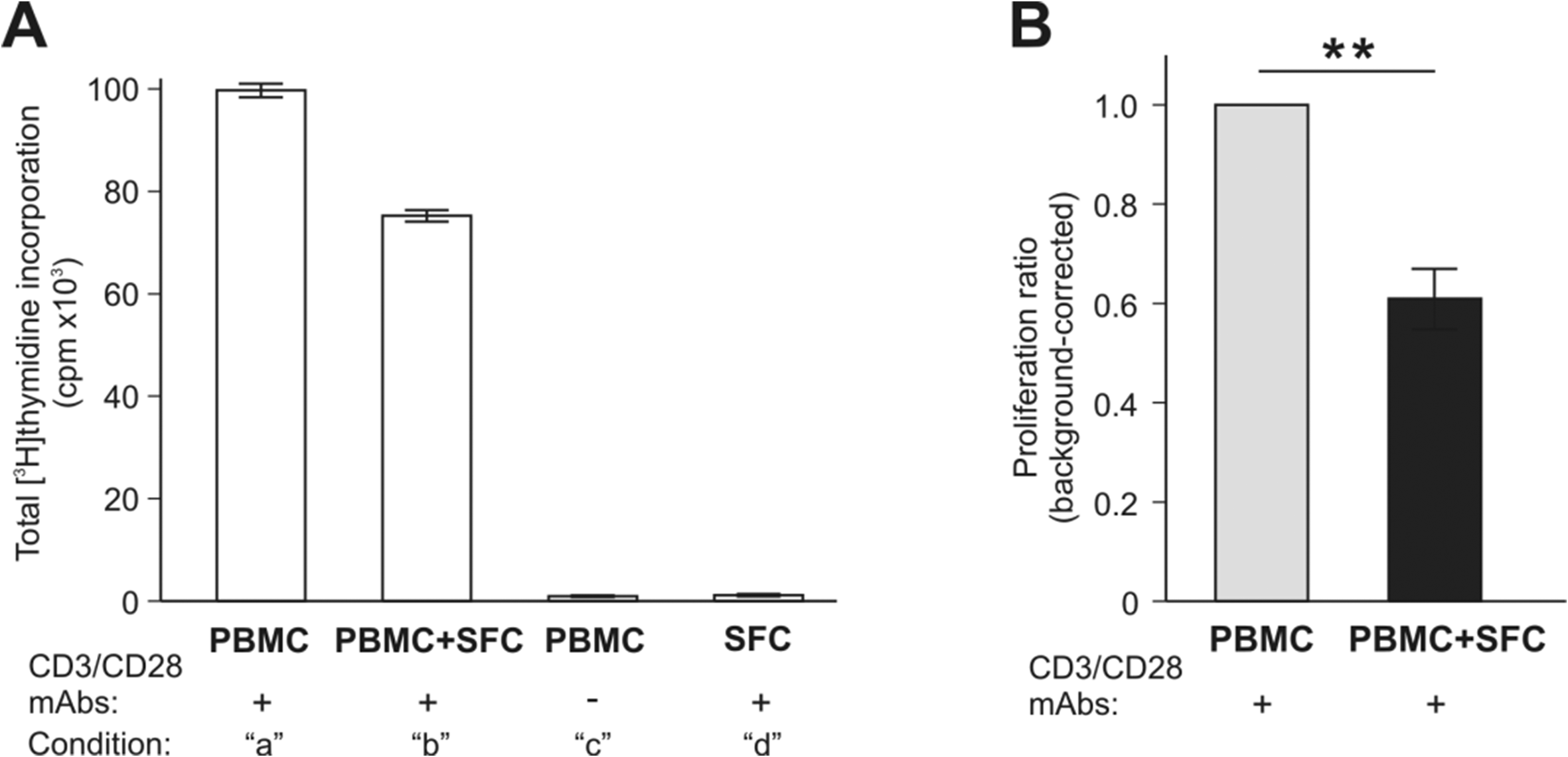

Supplement: Supplementary file 2 — Authors’ original file for figure 2 [file 12891_2014_2228_MOESM2_ESM.tif]

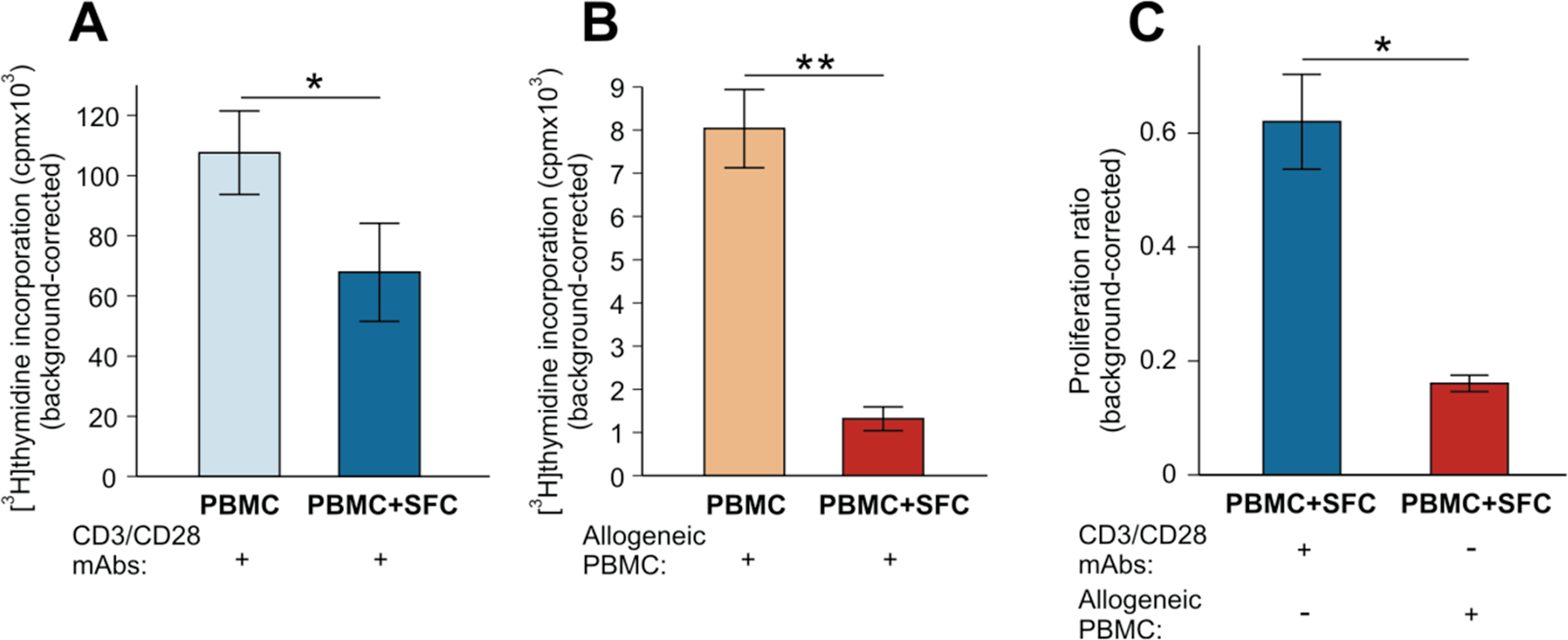

Supplement: Supplementary file 3 — Authors’ original file for figure 3 [file 12891_2014_2228_MOESM3_ESM.tif]
